# Adherence among HIV-positive injection drug users undergoing methadone treatment in Taiwan

**DOI:** 10.1186/s12888-020-02764-0

**Published:** 2020-07-02

**Authors:** En Chao, Chia-Chun Hung, Ching-Po Lin, Yi-Chien Jacob Ku, Qurat Ul Ain, David S. Metzger, Tony Szu-Hsien Lee

**Affiliations:** 1 Department of Health Promotion and Health Education, National Taiwan Normal University, Taipei, Taiwan; 2 Tri-Service General Hospital Songshan Branch, Taipei, Taiwan; 3 Institute of Brain Science, National Yang Ming University, Taipei, Taiwan; 4 Bali Psychiatric Center, Ministry of Health and Welfare, New Taipei City, Taiwan; 5 Department of Psychiatry, University of Pennsylvania, Philadelphia, USA; 6 Pir Mehr Ali Shah Arid Agriculture University, Rawalpindi, Pakistan; 7 CTBC Center for Addiction Prevention and Policy Research, National Taiwan Normal University, No 162 Sec. 1 He-Ping East Road, Taipei, 10610 Taiwan

**Keywords:** Adherence, Daily dose, Injection drug use, HIV, Methadone maintenance treatment, Retention

## Abstract

**Aims:**

The study aims were to investigate adherence to methadone maintenance treatment (MMT) and to identify associated clinical factors in patients who inject drugs diagnosed with human immunodeficiency virus (HIV) infection in Taiwan.

**Methods:**

Data were from the National Health Surveillance System on HIV and the National Drug Treatment System on MMT. HIV-positive people who inject drugs (HIVPWID) were defined as the study population. Information obtained included age, sex, education, marital status, employment, methadone dose, and date of diagnosis of HIV infection. Adherence was defined as taking methadone for the past 90, 180 and 365 days, then categorized as high (> 90%), moderate (51 to 90%), or low (<=50%) adherent respectively.

**Results:**

Of 1641 HIVPWID registered in the datasets from 2007 to 2012, 961 (58.56%) had received MMT. For HIVPWID evaluated at 90 days (*n* = 951), 271 (28.5%), 382 (40.2%), and 298 (31.3%) were classified as high, moderate, and low adherent respectively. For HIVPWID evaluated at 180 days (*n* = 936), 190 (20.3%), 349 (37.3%), and 397 (42.4%) were classified as high, moderate, and low adherent respectively. For HIVPWID evaluated at 365 days (*n* = 919), 133 (14.5%), 271 (29.5%), and 515 (56.0%) were classified as high, moderate, and low adherent respectively. After controlling for sociodemographics, results showed that methadone dose, location of MMT clinic, and date of HIV diagnosis were significantly associated with MMT adherence.

**Conclusions:**

Study findings underscore the importance to MMT adherence of methadone dosage, early diagnosis of patient’s HIV infection, and area of patient residence.

## Background

Opioid use disorder is a chronic relapsing disease defined by the loss or reduced control of the use of an opioid and expressed through persistent use despite the accumulation of negative consequences for health, social, financial, and family life [1]. Though people who inject drugs (PWID) can choose to stop addiction treatment to have a drug-free lifestyle based on their shared decision making with physicians, opioid addiction should be treated as a chronic disease that cannot be cured and requires long-term management [21].

PWID are often at high risk of contracting infectious diseases such as human immunodeficiency virus (HIV) and hepatitis C virus (HCV) [14]. In Taipei during the years 2009–2011, the prevalence rate of HIV and HCV infection among PWID at methadone centers was 17.7% [4] and 93% [14] respectively. To stem HIV and HCV transmission, methadone maintenance treatment (MMT) has been found to be effective in reducing needle sharing, frequency of injection, incidence of HIV infection, and reduction of criminal acts [6, 28, 32].

In efforts to improve MMT engagement and retention rates for HIV-positive individuals with opioid use disorder, a number of investigators have recommended the integration of MMT and antiretroviral therapy (ART) [12, 35]. Palepu et al. [24] showed that integrating ART and MMT resulted in better ART adherence rates, and Barker et al. [2] found that MMT was independently associated with optimal adherence to ART. These studies did not find negative impacts, such as ART discontinuation, by integrating it with MMT; instead, they reported a significant enhancement in plasma HIV RNA suppression among HIV-positive opioid-dependent drug users [2]. A recent study was conducted with men taking ART in Vietnam to compare those who also received MMT and those who did not. The results showed that the ART adherence rate was higher for men who received MMT. The authors pointed out that integrating MMT and ART was highly beneficial for improving adherence and reducing viral load and costs [25].

Recent research suggests that the retention rate in MMT is quite variable. Based upon a systematic review of 27 studies, MMT retention rates in China, which has the largest single network of programs globally, were found to be 89.4% (95% CI, 85.6–92.3%), 69.0% (57.7–78.4%), 62.9% (55.3–69.9%), 55.2% (48.5–61.7%) and 43.0% (34.7–51.7%) at 1, 3, 6, 12, and 24 months after admission, respectively [37]. Due to alcohol addiction and drug supplies in clubs, a poor adherence rate was reported in Guangzhou, China, where 21.0% of participants had dropped out of treatment and 27.7% revealed poor adherence during a period of 1 year [16]. With good promotion and awareness about MMT programs, adherence rates in South Asian countries, particularly Nepal, significantly improved, and 72.1% of participants were found to have high adherence to MMT, while only 9.1% had low adherence when observed for 1 month [29].

It is now well established that receiving an appropriate daily dose of methadone is critical to controlling withdrawal symptoms and reducing craving. In a randomized clinical trial, prescription of higher dosages minimized continued opioid use, and findings suggested that the minimum effective dose is around 60 mg/day [18]. Strain et al. [31] found that higher methadone doses were more effective than lower doses for patients with severe heroin use disorder.

Given the chronic nature of opioid use disorder, following treatment entry, both retention and adherence are necessary to optimize the impact of MMT on HIV prevention and care. PWID are prescribed methadone every day with adequate doses because the duration of drug effect on withdraw prevention extends for 24 h. Therefore, adherence to adequate dosages is critical because PWID who miss even a single daily dose will suffer withdrawal syndrome and are very likely to inject heroin (or other opioids) for symptom relief. Much research has focused on issues of retention in treatment, and it shows that the longer the duration of MMT, the better the outcome [6, 28, 32]. However, in comparison, limited attention has been focused on MMT adherence. The current study aimed to examine factors associated with adherence to MMT among HIV-positive patients who injected drugs (HIVPWID) at 90, 180, and 365 days after enrollment in MMT in Taiwan. It was hypothesized that higher daily methadone doses and treatment accessibility would be associated with better MMT adherence in HIV-positive PWID.

## Methods

### Ethics statement

This study protocol was reviewed and approved by the Research Ethics Committee at National Chang Hua Normal University (no. NCUEREC-103-013). All analyses utilized a de-identified national data set. Individual level consent was not possible nor was it determined not to be necessary by the Ethics Committee.

### Design and data selection

This is a retrospective study using data from the National Health Surveillance System on HIV voluntary counseling and testing and the National Drug Treatment System on MMT from 2007 to 2012. Information available and utilized included age, sex, education, marital status, employment, MMT location, daily methadone dosage, and date of HIV infection if known. Data were obtained for 1641 HIVPWIDs for whom we were able to match methadone treatment records in the National Drug Treatment System. This resulted in 574,024 data points after removing duplicate and missing records. Information regarding the patients’ antiretroviral therapy treatment was not available.

### Outcome variables

Adherence was the main dependent variable in this study. Previous research has classified greater than 90% adherence as “high”, 51–90% adherence as “moderate”, and less than or equal to 50% as "low [38]. In this study, patients’ adherence to MMT was determined at three time points (90 days, 180 days, and 365 days after treatment onset) based on daily attendance. For example, a patient taking methadone for 82 of 90 days was categorized as having high adherence (82/90 = 91%). MMT adherence was defined as attendance days divided by total days across the times.

$$MMT\; adherence\kern0.17em rate=\frac{Days\kern0.17em of\kern0.17em attendance\kern0.17em during\kern0.17em the\; MMT\; period}{Days\kern0.17em of\kern0.17em specified\; MMT\; period\;\left(90,180\; and\;365\; days\right)}$$

### Independent variables

Demographic variables were age, sex, education, marital status, and employment. The three independent variables included in this analysis were methadone dose, location of MMT program, and duration of HIV infection. Previous studies have demonstrated that a methadone dose of ≥60 mg is most effective in achieving positive MMT outcomes [5, 30, 31, 38]. To allow for examination of the impact of differences in methadone dosage, we divided the HIVPWID into two groups. The first group received less than 60 mg per day and the second group received 60 mg or more per day.

We measured the differences in MMT administration and programming for urban vs. rural areas [13]. The geographic locations included were Taipei metro area, Kaohsiung metro area, and Taiwan County. There are 7.07, 3.91, and 2.64 MMT clinics per 10,000 km^2^ in Taipei, Kaohsiung, and Taiwan County respectively.

It is well established that HIV infection is associated with a range of psychopathologies due to the receipt of an HIV positive diagnosis when assessed at 180 days of follow-up evaluation [3, 23]. To examine the impact of the known duration of HIV infection, we divided the patients into three groups. The first group was diagnosed HIV-positive more than 180 days before attending MMT, the second group was diagnosed HIV-positive less than 180 days before attending MMT, and the third group was diagnosed HIV-positive only after attending MMT.

### Analysis

Descriptive analyses were performed using ANOVA for continuous variables and chi-square for categorical variables. Ordered logistic regression was used to examine the factors associated with MMT adherence, because adherence was categorized into 3 groups. Time varying analysis was not performed because one of the proposed hypotheses was to examine if a 60 mg methadone dose was associated with adherence. One of the assumptions underlying ordered logistic regression is that the relationship between each pair of outcome groups is the same. The results of an approximate likelihood-ratio test and a Brant test indicated that the proportional odds assumption was not violated. Analyses were conducted using SAS Version 9.4 (SAS Institute Inc., Cary, NC, USA) and STATA 14.2. The significance criterion was set at 0.05.

## Results

Of 1641 HIVPWID during 2007 to 2012 identified in Taiwan, 961 (58.56%) attended MMT. Patient characteristics stratified by MMT adherence over 90 days, 180 days, and 365 days are presented in Table 1. For HIVPWID evaluated at 90 days (*n* = 951, 98.9%), 271 (28.5%), 382 (40.2%), and 298 (31.3%) were classified as having high, moderate, and low adherence to MMT, respectively. For HIVPWID evaluated at 180 days (*n* = 936; 97%), 189 (20.2%), 350 (37.4%), and 397 (42.4%) were categorized as high, moderate, and low adherence to MMT, respectively. For HIVPWID evaluated at 365 days (*n* = 919; 95.7%), 132 (14.4%), 271 (29.5%), and 516 (56.1%) were categorized as high, moderate, and low adherence to MMT, respectively.

**Table 1:** Characteristics of HIV-positive (HIV+) PWIDs stratified by 90, 180, and 365 days of MMT adherence

*Characteristics*	*Baseline*^*a*^ *n = 961*	90 days (*n* = 951; 98.9%)			*p*^*b*^	180 days (*n* = 936; 97.4%)			*p*^*b*^	365 days (*n* = 919; 95.6%)			*p*^*b*^
		*Low* *n = 298*	*Moderate* *n = 382*	*High* *n = 271*		*Low* *n = 397*	*Moderate* *n = 350*	*High* *n = 189*		*Low* *n = 516*	*Moderate* *n = 271*	*High* *n = 132*	
Age, mean (sd)	37.40 (8.34)	36.60 (7.72)	37.09 (8.39)	38.76 (8.75)	.005^c^	36.66 (7.96)	37.35 (8.13)	38.97 (9.10)	.007^d^	36.78 (7.89)	37.37 (8.45)	39.42 (9.25)	.005^e^
Gender, n(%)					.098				.663				.663
Female	137	53 (39)	49 (36)	33 (24)		59 (44)	51 (38)	23 (17)		74 (56)	42 (32)	16 (12)	
Male	824	245 (30)	333 (41)	238 (29)		338 (42)	299 (37)	166 (21)		442 (56)	229 (29)	116 (15)	
Education level, n(%)					.012				.003				.034
< 9 years	679	230 (34)	264 (39)	180 (27)		304 (46)	236 (36)	122 (19)		376 (58)	193 (30)	81 (13)	
> =9 years	282	68 (25)	118 (43)	91 (33)		93 (34)	114 (42)	67 (25)		140 (52)	78 (29)	51 (19)	
Marital status, n(%)					.524				.091				.041
Married	111	35 (32)	46 (42)	28 (26)		44 (41)	37 (35)	26 (24)		50 (47)	33 (31)	23 (22)	
Married but separated	61	21 (34)	27 (44)	13 (21)		33 (55)	19 (32)	8 (13)		38 (64)	16 (27)	5 (9)	
Single	510	167 (33)	196 (39)	143 (28)		215 (43)	194 (39)	88 (18)		290 (60)	133 (27)	62 (13)	
Divorced	279	75 (27)	113 (41)	87 (32)		105 (39)	100 (37)	67 (25)		138 (51)	89 (33)	42 (16)	
Employment status, n(%)					.006				.004				.013
Not employed	568	194 (35)	228 (41)	141 (25)		249 (45)	214 (39)	92 (17)		324 (60)	155 (29)	65 (12)	
Employed	393	104 (27)	154 (40)	130 (34)		148 (39)	136 (36)	97 (26)		192 (51)	116 (31)	67 (18)	
Case management city, n(%)					<.001				<.001				<.001
Taipei metro area	315	71 (23)	134 (43)	109 (35)		100 (33)	120 (39)	88 (29)		139 (46)	96 (32)	68 (22)	
Kaohsiung metro area	330	108 (33)	123 (38)	95 (29)		145 (45)	120 (37)	56 (17)		188 (60)	97 (31)	28 (9)	
Taiwan County	316	119 (38)	125 (40)	67 (22)		152 (50)	110 (36)	45 (15)		189 (62)	78 (26)	36 (12)	
MMT mean dosage, n(%)					<.001				<.001				<.001
< 60 mg	512	274 (35)	302 (39)	198 (26)		346 (47)	263 (36)	132 (18)		421 (60)	197 (28)	90 (13)	
> =60 mg	107	24 (14)	80 (45)	73 (41)		51 (26)	87 (45)	57 (29)		95 (45)	74 (35)	42 (20)	
HIV+ diagnosed & in MMT, n(%)					.073				.042				.002
HIV+ before MMT > = 180 days	437	126 (27)	195 (42)	141 (31)		169 (37)	183 (41)	100 (22)		218 (50)	129 (34)	69 (16)	
HIV+ before MMT < 180 days	175	56 (32)	71 (40)	50 (28)		81 (46)	58 (33)	36 (21)		112 (64)	36 (21)	27 (15)	
HIV+ after MMT	312/309/307	116 (37)	116 (37)	80 (26)		147 (48)	109 (35)	53 (17)		186 (61)	85 (28)	36 (12)	

^a^Baseline MMT mean dosage calculated from the first 30 days

^b^*p* value for difference between low, moderate, and high groups

^c^Post hoc comparison: high > low, moderate > low by Dunnett’s T3 method

^d^Post hoc comparison: high > low by Dunnett’s T3 method

^e^Post hoc comparison: high > low by Dunnett’s T3 method

The ANOVA/chi-square analyses examining participant characteristics associated with adherence at 90, 180, and 365 days are reported in Table 1. Participants with high adherence were older (mean = 38.76 years, *p* < .01) and more likely to have more than 9 years of education (*p* < .05). Gender was not significantly associated with retention at any of the time points. Higher rates of adherence were observed among those who were married, but this relationship was significant (*p* < .05) only at the 365-day assessment.

Employment was significantly (*p* < .05) associated with adherence at each time point. Regional differences were significant at each time point. Those patients receiving treatment in Taipei were more likely (*p* < .001) to be in the high adherence group for each time interval. HIVPWID with mean daily methadone dosages of ≥60 mg/day were significantly (*p* < .001) more likely to be in the high adherence group at each assessment point. Adherence was higher at 180 and 365 days for those patients who were informed of their HIV status 180 days before entering MMT.

The results of the ordered logistic regression analyses are presented in Table 2. Both the Brant Test of Parallel Regression Assumption and the approximate likelihood ratio chi-square test indicate that the proportional odds assumption was not violated. Table 2 shows age (older), education (≥9 years), marital status (married) and employment are protective factors for MMT adherence.

**Table 2:** Ordered logistic regression on adherence for 90, 180, and 365 days of MMT^a^

	90 days ^b^			180 days ^c^			365 days ^d^		
*Characteristics*	*OR*	*95% CI*		*OR*	*95% CI*		*OR*	*95% CI*	
Age	1.02**	1.01	1.04	1.02**	1.01	1.04	1.02**	1.01	1.04
Gender									
Female (RC)	1.00			1.00			1.00		
Male	1.41	0.97	2.06	1.20	0.82	1.74	1.11	0.75	1.64
Education level									
< 9 years (RC)	1.00			1.00			1.00		
> =9 years	1.51**	1.15	1.97	1.65***	1.26	2.16	1.44*	1.09	1.92
Marital status									
Married (RC)	1.00			1.00			1.00		
Married but separated	0.73	0.40	1.31	0.48*	0.26	0.89	0.38**	0.20	0.74
Single	0.93	0.62	1.40	0.77	0.51	1.17	0.55**	0.36	0.84
Divorced	1.27	0.84	1.93	1.10	0.71	1.68	0.76	0.49	1.18
Employment status									
Not employed (RC)	1.00			1.00			1.00		
Employed	1.36*	1.06	1.75	1.24	0.96	1.60	1.28	0.98	1.68
Case management area									
Taipei metro area (RC)	1.00			1.00			1.00		
Kaohsiung metro area	0.76	0.56	1.02	0.61**	0.45	0.83	0.55***	0.40	0.75
Taiwan County	0.59**	0.44	0.80	0.56***	0.41	0.77	0.57**	0.41	0.79
MMT mean dosage									
< 60 mg (RC)	1.00			1.00			1.00		
> =60 mg	2.58***	1.89	3.52	2.18***	1.62	2.95	1.80***	1.33	2.43
HIV+ diagnosed & in MMT date									
HIV+ before MMT > = 180 days (RC)	1.00			1.00			1.00		
HIV+ before MMT < 180 days	0.82	0.59	1.14	0.70*	0.50	0.98	0.55**	0.38	0.79
HIV+ after MMT	0.65**	0.50	0.86	0.61**	0.46	0.81	0.58***	0.43	0.78

Notes: *OR* odds ratio, *CI* confidence interval, *RC* reference category

^a^Adherence of 90 days, 180 days, and 365 days for MMT ordered by > = 90%, 50–90, < 50%

^b,c,d^ Brant Tests of Parallel Regression Assumption were non-significant (*p* > .05) and approximate likelihood-ratio test of odds across response categories were non-significant (*p* > .05)

*p < 0.05; **p < 0.01; ***p < 0.001

There were significant adherence differences between patients receiving services in the Taipei metro area and patients treated in other regions. Especially for those patients receiving services from the Taiwan county region, the odds of achieving high adherence were significantly lower at 90, 180 and 365 days (90 day odds ratio (OR)) = 0.59, 95% CI: 0.44–0.80; 180 day OR = 0.56, 95% CI: 0.41–0.77; 365 day OR = 0.57, 95% CI: 0.41–0.79). With regard to dosage, the model indicates that with mean dosages ≥60 mg, the odds of achieving high adherence were significantly increased (90 day OR = 2.58, 95% CI, 1.89–3.52, 180 day OR = 2.18, 95% CI: 1.62–2.95, and 365 day OR = 1.80, 95% CI: 1.33–2.43).

In examining the timing of HIV diagnosis, we found significantly lower rates of adherence at 180 and 365 days among those diagnosed less than 180 days before initiating MMT (180 day OR = 0.70, 95% CI: 0.50–0.98, and 365 day OR = 0.55, 95% CI: 0.38–0.79).

## Discussion

This analysis is unique because it looks at the proportion of completed MMT medication visits. To our knowledge, this may be the largest study of its type studying HIV-positive patients. The analyses reported here document a very high rate of retention in MMT among PWID living with HIV in Taiwan. Over 90% of all who entered treatment continued in treatment for at least 1 year. One plausible explanation is that all PWID living with HIV were encouraged to attend MMT and ART with all expenses paid by the Taiwanese government.

While retention rates were high, the consistent completion of medication visits which indicate adherence, is perhaps a more important measure of treatment engagement and efficacy. The quantification of adherence to MMT represents a major contribution of these analyses and allows for a careful examination of factors that were associated with completion of medication administration visits. Our findings specify that 28.50, 20.30, and 14.47% patients were very highly adherent for 90 days, 180 days, and 365 days respectively. A similar trend was found in a study conducted in France by Roux et.al in 2014, in which adherence rates were recorded for 365-day periods [27]. However, the adherence rates in our study revealed a substantial gap that could be improved, which could result in an increase in the number of medication visits by HIVPWID.

As hypothesized, adherence was strongly associated with methadone dosages of 60 mg per day or higher. Low-dose methadone (less than 60 mgs/day) has been shown to have limited effectiveness, whereas treatment with higher dosages is associated with improved treatment outcomes such as reductions in heroin use and increased retention rates [20]. A study by Donny [5] indicates that the frequency of heroin injections was inversely proportional to the methadone dosage. In this study, three different dosages (50, 100 and 150 mg) were selected for treatment and the results showed that the higher dosage resulted in better control of the subjective effects and effective cross-tolerance to heroin. However, with a low dose (50 mg), alternative monetary reinforcement was needed to control the heroin use, which was not the case with a high (150 mg) dose of methadone [5]. Later, it was found that the appropriate methadone dosage to be highly adherent ranged from 80 to 150 mg [8, 10, 22].

As hypothesized, and consistent with previous research, our data document a strong association between dosage and adherence. Marienfeld et al. [17] and Shen et al. [30] found that lower dosages of methadone were significantly associated with low or poor adherence. According to WHO guidelines, the minimum recommended dose of methadone is 60 mg/day, while a majority of patients require up to 120 mg/day [36]. However, it has been found that in many Asian countries, the dosage is still below these standards, although there are limited data on the exact methadone dosage used during MMT [26]. One study reported that in Malaysia, 40 mg/day is the minimum dose required to help prevent re-injection habits during MMT therapy, and the authors reported that achieving the best outcome with MMT required a minimum of 80 mg/day [26]. However, only 5% of patients are receiving at least 40 mg/day in Malaysia and the average methadone dose is just 10–20 mg/day [7]. Similarly, some clinics in Thailand (Bangkok) provide lower doses of methadone (< 60 mg/day) to patients [11]. It is reported that in Wuhan, China, most PWID are prescribed a methadone dose of more than 60 mg/day, but only 18% of patients are taking the recommended dose (≥ 60 mg/day) and the majority of patients are not receiving a high enough dose. In spite of the efficiency and success of methadone treatment, other issues are still present, such as homelessness, unemployment, lack of family or friend support, and other treatment complications [17]. A matched study of Vietnamese patients highlights that the role of family support and having a mobile phone can play a significant supporting role in enhancing high adherence to MMT [34].

This study’s results show that the patients who were diagnosed with HIV 180 days before enrolling in MMT had better MMT adherence than those diagnosed more recently before initiating MMT. This finding is important because early suppression of the virus is important for both personal and public health. Most clinical guidelines recommend immediate initiation of ART to suppress the virus and prevent transmission. Learning of one’s HIV diagnosis, like that of any life-threatening illness, can be traumatic. The Kübler-Ross Grief Cycle [9] characterizes the progression of the emotional experience in response to HIV/AIDS as moving from denial to anger, depression, bargaining, and finally to acceptance. People who learn of their HIV diagnosis do not necessarily go through the stages in the same order or experience all of them, but they may go through a period of time adjusting themselves through denial, anger, and depression. During this period, HIVPWID may not actively engage in MMT and ART as they focus on their emotional distress and disturbance. Our findings may point to the fact that this period for HIVPWID lasts about 180 days. Second, HIV associated stigma is prevalent within the PWID population. This often causes PWID to avoid treatment in order not to be recognized as such in health care settings. In Taiwan, HIV testing and counseling must be confidential, but attending appointments at an HIV clinic may cause HIV-positive individuals to fear exposing their HIV status. The data from this study cannot explain why those with recent diagnoses were the most likely to have low MMT adherence. Future research is needed to understand what may influence this.

Our results found that MMT adherence is also associated with the location of the patient’s MMT center [33]. Patients served by programs within the Taipei metropolitan area were found to have better adherence than those receiving services from programs located in Kaohsiung and Taiwan County. People living in metropolitan Taipei have easier access to facilities because of more MMT clinics per square km, hospitals, and other resources. A matched study by Groh et al. reported similar results to ours with lower MMT adherence rates in rural areas than in urban areas or developed cities, which they attributed to lack of facilities, poor access to care, and poor education and health systems [9]. An earlier study conducted in Taiwan among newly admitted MMT patients found that the distance between home and the MMT clinic was associated with duration of MMT treatment [15]. In other related studies, adherence rates were found to be lower in Africa than Western countries. Studies of poor resource settings in Senegal and sub-Saharan Africa have led to the assumption that low adherence on the continent is caused by several Africa-specific factors, particularly the high cost of medicines and the lack of health infrastructure [19].

### Limitations

Although this study has strength in utilizing national health data, it has limitations. ART status is an indicator of medical treatment engagement and thus has an impact on MMT treatment adherence. ART records are not included in the analysis, but must be considered because ART usage might influence MMT dosage due to potential drug interactions. The well-established relationship between MMT treatment adherence and ART success supports the critical importance of MMT adherence, yet without the linkage to data from ART programming, the impact of MMT cannot be confirmed. Lastly, we did not take patients’ everyday dose utilized with time-varying model. Instead, the average dose for 90, 180 and 365 days was calculated to determine if a patient took more than 60 mg methadone in this study.

## Conclusion

In our study we found that being older, better educated, and married; taking 60 mg or more of methadone per day; living in the Taipei metropolitan area; and learning of one’s HIV status more than 180 days were associated with high MMT adherence among our sample. The findings on clinical dosing, geographic variability, and poor adherence to MMT shortly after diagnosis of HIV infection are critical. These findings highlight the need for scientific implementation to follow clinical guidelines, the need to improve the medical education of MMT providers, and the need for future research linking MMT to ART data.

## Data Availability

Data are available from the corresponding author, upon reasonable request..

## Abbreviations

HIV: Human immunodeficiency virus
PWID: People who inject drugs
MMT: Methadone maintenance treatment
HIVPWID: HIV positive people who inject drugs

## References

[1] Auriacombe M, Moriceau S, Serre F, Denis C, Micoulaud-Franchi JA, de Sevin E, Bonhomme E, Bioulac S, Fatseas M, Philip P (2018). Development and validation of a virtual agent to screen tobacco and alcohol use disorders. Drug Alcohol Depend.

[2] Barker B, Adams E, Wood E, Kerr T, DeBeck K, Dong H, Shoveller J, Montaner J, Milloy MJ (2019). Engagement in maximally-assisted therapy and adherence to antiretroviral therapy among a cohort of indigenous people who use illicit drugs. AIDS Behav.

[3] Brief D, Bollinger J, Vielhauer AR, Berger-Greenstein MJ, Morgan JA, Brady EE, Buondonno SM, Keane LM, T. M. & For The Hiv/aids Treatment Adherence, Health Outcomes And Cost Study Group (2004). Understanding the interface of HIV, trauma, post-trauMMTic stress disorder, and substance use and its implications for health outcomes. AIDS Care.

[4] Chou YC, Shih SF, Tsai WD, Li CS, Xu K, Lee TS (2013). Improvement of quality of life in methadone treatment patients in northern Taiwan: a follow-up study. BMC Psychiatry.

[5] Donny EC, Brasser SM, Bigelow GE, Stitzer ML, Walsh SL (2005). Methadone doses of 100 mg or greater are more effective than lower doses at suppressing heroin self-administration in opioid-dependent volunteers. Addiction.

[6] Dvoriak S, Karachevsky A, Chhatre S, Booth R, Metzger D, Schumacher J, Chychula N, Pecoraro A, Woody G (2014). Methadone maintenance for HIV positive and HIV negative patients in Kyiv: acceptability and treatment response. Drug Alcohol Depend.

[7] Fanoe S, Hvidt C, Ege P, Jensen GB (2007). Syncope and QT prolongation among patients treated with methadone for heroin dependence in the city of Copenhagen. Heart.

[8] Gowing L, Farrell MF, Bornemann R, Sullivan LE, Ali R. Oral substitution treatment of injecting opioid users for prevention of HIV infection. Cochrane Database of SysteMMTic Reviews. 2011;8:Cd004145. 10.1002/14651858.CD004145.pub3 18425898

[9] Groh K, Audet CM, Baptista A, Sidat M, Vergara A, Vermund SH, Moon TD (2011). Barriers to antiretroviral therapy adherence in rural Mozambique. BMC Public Health.

[10] Gronbladh L, Ohlund LS, Gunne LM (1990). Mortality in heroin addiction: impact of methadone treatment. Acta Psychiatr Scand.

[11] Hayashi K, Ti L, Ayutthaya PPN, Suwannawong P, Kaplan K, Small W, Kerr T (2017). Barriers to retention in methadone maintenance therapy among people who inject drugs in Bangkok, Thailand: a mixed-methods study. Harm Reduct Journal.

[12] Jiang H, Cao X, Wang C, Luo W, Li J, Rou K, Zhang B, Fang Y, Li C, Wu Z (2014). Study on the adherence and related determinants among HIV-positive clients under methadone maintenance treatment in Dali,Yunnan province from 2005 to 2013. Zhonghua Liu Xing Bing Xue Za Zhi.

[13] Kourounis G, Richards BD, Kyprianou E, Symeonidou E, Malliori MM, Samartzis L (2016). Opioid substitution therapy: lowering the treatment thresholds. Drug Alcohol Depend.

[14] Lee TS, Shen HC, Wu WH, Huang CW, Yen MY, Wang BE, Chuang P, Shih CY, Chou YC, Liu YL (2011). Clinical characteristics and risk behavior as a function of HIV status among heroin users enrolled in methadone treatment in northern Taiwan. Substance Abuse Treatment Prevention Policy.

[15] Lin CK, Hung CC, Peng CY, Chao E, Lee TS (2015). Factors associated with methadone treatment duration: a cox regression analysis. PLoS One.

[16] Liu D, Gu J, Xu H, Hao C, Jiao M, Zhang X, Zhao Y, Andrew B, Hao Y (2017). Club drugs and alcohol abuse predicted dropout and poor adherence among methadone maintenance treatment patients in Guangzhou, China. AIDS Care.

[17] Marienfeld C, Liu P, Wang X, Schottenfeld R, Zhou W, Chawarski MC (2015). Evaluation of an implementation of methadone maintenance treatment in China. Drug Alcohol Depend.

[18] Maxwell S, Shinderman M (1999). Optimizing response to methadone maintenance treatment: use of higher-dose methadone. J Psychoactive Drugs.

[19] McCance-Katz EF (2005). Treatment of opioid dependence and coinfection with HIV and hepatitis C virus in opioid-dependent patients: the importance of drug interactions between opioids and antiretroviral agents. Clin Infect Dis.

[20] McGlothlin WH, Anglin MD (1981). Shutting off methadone. Costs and benefits. Arch Gen Psychiatry.

[21] McLellan AT, Lewis DC, O'Brien CP, Kleber HD (2000). Drug dependence, a chronic medical illness: implications for treatment, insurance, and outcomes evaluation. JAMA.

[22] Newman RG, Whitehill WB (1979). Double-blind comparison of methadone and placebo maintenance treatments of narcotic addicts in Hong Kong. Lancet.

[23] Olley B, Seedat S, Stein DJ (2006). Persistence of psychiatric disorders in a cohort of HIV/AIDS patients in South Africa: a 6-month follow-up study. J PsychosoMMTic Res.

[24] Palepu A, Tyndall MW, Joy R, Kerr T, Wood E, Press N, Hogg RS, Montaner JS (2006). Antiretroviral adherence and HIV treatment outcomes among HIV/HCV co-infected injection drug users: the role of methadone maintenance therapy. Drug Alcohol Depend.

[25] Reddon H, Milloy MJ, Simo A, Montaner J, Wood E, Kerr T (2014). Methadone maintenance therapy decreases the rate of antiretroviral therapy discontinuation among HIV-positive illicit drug users. AIDS Behav.

[26] Reid G, Sharma M, Higgs P (2014). The long winding road of opioid substitution therapy implementation in South-East Asia: challenges to scale up. J Public Health Res.

[27] Roux P, Lions C, Michel L, Cohen J, Mora M, Marcellin F, Spire B, Morel A, Carrieri PM, Karila L (2014). Predictors of non-adherence to methadone maintenance treatment in opioid-dependent individuals: implications for clinicians. Curr Pharm Des.

[28] Russolillo A, Moniruzzaman A, McCandless LC, Patterson M, Somers JM (2018). Associations between methadone maintenance treatment and crime: a 17-year longitudinal cohort study of Canadian provincial offenders. Addiction.

[29] Sharma V, Chamroonswasdi K, Srisorrachatr S (2016). Rate of adhernce to and factors associated with methadone maintenance treatment program (MMTP) compliance among injecting drug use patients in Nepal. Southeast Asian J Trop Med Public Health.

[30] Shen J, Wang M, Wang X, Zhang G, Guo J, Li X, Li J (2016). Predictors of poor adherence to methadone maintenance treatment in Yunnan Province, China. J Addict Med.

[31] Strain EC, Bigelow GE, Liebson IA, Stitzer ML (1999). Moderate- vs high-dose methadone in the treatment of opioid dependence: a randomized trial. JAMA.

[32] Sullivan LE, Metzger DS, Fudala PJ, Fiellin DA (2005). Decreasing international HIV transmission: the role of expanding access to opioid agonist therapies for injection drug users. Addiction.

[33] Tran BX, Nguyen LH, Tran TT, Latkin CA (2018). Social and structural barriers for adherence to methadone maintenance treatment among Vietnamese opioid dependence patients. PLoS One.

[34] Van Nguyen H, Nguyen HL, Mai HT, Le HQ, Tran BX, Hoang CD, Le HT, Nguyen CT, Tran TD, Latkin CA, Vu TM (2017). StigMMTization among methadone maintenance treatment patients in mountainous areas in northern Vietnam. Harm Reduct J.

[35] Volkow ND, Montaner J (2011). The urgency of providing comprehensive and integrated treatment for substance abusers with HIV. Health Aff.

[36] World Health Organization, Regional Office for South-East Asia (2008). Operational guidelines for the management of opioid dependence in the South-East Asia region.

[37] Zhang L, Chow EP, Zhuang X, Liang Y, Wang Y, Tang C, Ling L, Tucker JD, Wilson DP (2013). Methadone maintenance treatment participant retention and behavioural effectiveness in China: a systeMMTic review and meta-analysis. PLoS One.

[38] Zhou K, Li H, Wei X, Li X, Zhuang G (2017). Medication adherence in patients undergoing methadone maintenance treatment in Xi'an, China. J Addict Med.

