# Correction to: Adherence among HIV-positive injection drug users undergoing methadone treatment in Taiwan

**DOI:** 10.1186/s12888-021-03161-x

**Published:** 2021-03-23

**Authors:** En Chao, Chia-Chun Hung, Ching-Po Lin, Yi-Chien Jacob Ku, Qurat Ul Ain, David S. Metzger, Tony Szu-Hsien Lee

**Affiliations:** 1 Department of Health Promotion and Health Education, National Taiwan Normal University, Taipei, Taiwan; 2 Tri-Service General Hospital Songshan Branch, Taipei, Taiwan; 3 Institute of Brain Science, National Yang Ming University, Taipei, Taiwan; 4 Department of Psychiatry, University of Pennsylvania, Philadelphia, USA; 5 Department of Psychiatry, University of Pennsylvania, Philadelphia, USA; 6 Pir Mehr Ali Shah Arid Agriculture University, Rawalpindi, Pakistan; 7 CTBC Center for Addiction Prevention and Policy Research, National Taiwan Normal University, No 162 Sec. 1 He-Ping East Road, Taipei, 10610 Taiwan

**Correction to: BMC Psychiatry (2020) 20:346**

**https://doi.org/10.1186/s12888-020-02764-0**

Following publication of the original article [1], the authors identified an error in the affiliation of Dr. David S. Metzger.

The incorrect affiliation: Bali Psychiatric Center, Ministry of Health and Welfare, New Taipei City, Taiwan.

The correct affiliation: Department of Psychiatry, University of Pennsylvania, Philadelphia, USA.

The author group has been updated above and the original article [1] has been corrected.
